# Efficacy of Combination Docetaxel and Nintedanib in Advanced Non-Small Cell Lung Cancer in Thailand: A Multicenter Study

**DOI:** 10.3389/fonc.2021.572740

**Published:** 2021-04-29

**Authors:** Krittiya Korphaisarn, Pongwut Danchaivijitr, Thanyanan Reungwetwattana, Busayamas Chewaskulyong, Luangyot Thongthieang, Jarin Chindaprasirt, Kunlatida Maneenil, Chirawadee Sathitruangsak, Chanida Vinayanuwattikun

**Affiliations:** ^1^ Department of Medicine, Faculty of Medicine Siriraj Hospital, Mahidol University, Bangkok, Thailand; ^2^ Department of Medicine, Faculty of Medicine Ramathibodi Hospital, Mahidol University, Bangkok, Thailand; ^3^ Department of Medicine, Faculty of Medicine, Chiangmai University, Chiang Mai, Thailand; ^4^ Department of Medicine, Faculty of Medicine, Khon Kaen Hospital. Khon Kaen University, Khon Kaen, Thailand; ^5^ Department of Medicine, Faculty of Medicine, Srinagarind Hospital, Khon Kaen University, Khon Kaen, Thailand; ^6^ Department of Medicine, Rajavithi Hospital, Bangkok, Thailand; ^7^ Holistic Center for Cancer Study and Care (HOCC-PSU) and Department of Medicine, Faculty of Medicine, Prince of Songkla University, Songkhla, Thailand; ^8^ Department of Medicine, Faculty of Medicine, Chulalongkorn University and The King Chulalongkorn Memorial Hospital, Bangkok, Thailand

**Keywords:** docetaxel, nintedanib, non-small cell lung cancer, sequential treatment, anti-angiogenesis therapy

## Abstract

**Introduction:**

The mainstay systemic treatment for non-oncogenic addictive advanced stage non-small cell lung cancer is chemotherapy. Anti-angiogenic agents are additive compounds that enhance disease control and lead to improvement of overall survival benefit. Recently PD-(L)1 blockage, a checkpoint inhibitor, has been adopted as another line of treatment. A sequential strategy to enhance the efficacy of combination docetaxel and nintedanib after immunotherapy, correlated with genomic mutation, has been explored.

**Method:**

A retrospective cohort study of 56 patients from 8 centers in Thailand who received combination docetaxel and nintedanib *via* the Thai nintedanib Named Patient Use program was conducted. Demographic characteristics, treatment details, and treatment responses were retrieved from medical records.

**Results:**

The majority of patients were male (62.5%) with adenocarcinoma subtype (88%). Thirty-five percent had sensitizing *EGFR* mutation. Combination docetaxel and nintedanib was given as second to fourth line of treatment. Median PFS of docetaxel/nintedanib was 5.6 months [95% CI 4.8-6.9]. Median OS of the entire cohort was 22.5 months [95% CI 20.2-31.1]. Among them, only four patients received this combination after immunotherapy which limited the validity of efficacy analysis. Median PFS of those four patients was 7.9 months [range 5.2-9.1] which was slightly higher than the remaining cohort (median PFS 4.5 months, 95% CI: 4.0-6.0, *p*-value 0.09). Among the adenocarcinoma subtype, a relapse-time of platinum-doublet chemotherapy of more than 6 months was solely indicated as a benefit of combination docetaxel/nintedanib treatment compared to the relapse-time of platinum-doublet chemotherapy of less than 6 months by multivariate HR of PFS 0.32 [95% CI: 0.14-0.68, *p*-value 0.003].

**Conclusion:**

Combination docetaxel and nintedanib provided more benefit in relapse-time of platinum-doublet chemotherapy of more than 6 months in advanced stage adenocarcinoma lung cancer. Neither *EGFR* nor *ALK* alteration influenced the outcome of treatment.

## Introduction

The development of current standard treatments of advanced non-small cell lung cancer has led to the improvement of survival outcome. Novel strategies adopting predictive biomarkers have guided treatment towards an era of precision medicine. Biomarker discoveries to define more targeted therapies have been explored in many clinical studies. For non-targetable advanced non-small cell lung cancer, which has no targeted therapy option, there seems to be fewer treatment opportunities and worse prognosis outcome (1). Combination antiangiogenic therapies and chemotherapy has improved the efficacy of treatment in non-small cell carcinoma lung cancer by normalizing abnormal tumor vasculature and enhancing tumor shrinkage. A combination of docetaxel/nintedanib was approved by the USFDA as a subsequent treatment after platinum-resistance in advanced adenocarcinoma lung cancer patients. Significant improvement in progression-free survival (PFS) with a median of 3.4 months vs. 2.7 months compared to placebo and docetaxel has been shown in the phase III LUME-lung 1 global study (2). Furthermore, PD-(L)1 blockage, a novel immunotherapy, has shown benefits for improving survival outcomes, either by monotherapy or combination with chemotherapy (3–5). Chemotherapy enhances the effect of immunotherapy by increasing recognition, eliminating tumor cells by the host immune system, and reducing the immunosuppressive microenvironment (6). Furthermore, preclinical reports revealed that VEGFR blockage inhibits suppressive immune cells (MDSC, Treg, macrophages) and increases mature dendritic cell results in delayed tumor growth (7, 8). Combination anti-angiogenic and PD-(L)1 blockage has shown significant tumor control (9). Sequence of immunotherapy before subsequent docetaxel/nintedanib treatment has also shown improved response to treatment in a retrospective cohort (10, 11).

The Thai non-squamous cell carcinoma of the lung has up to 57% predominated *EGFR* mutation (12), contrary to the Western non-squamous lung cancer population, which has less than 10% prevalence of *EGFR* mutation. Comparing the efficacy in our country to a global study that enrolled a majority of Caucasian patients might help us to understand the real benefits of treatment of Asian patients. We report a retrospective cohort study of advanced stage non-small lung cancer patients who received subsequent treatment of docetaxel/nintedanib after platinum-resistant advanced stage lung cancer to explore treatment efficacy in terms of *EGFR/ALK* alteration status and efficacy of treatment following immunotherapy.

## Materials and Methods

### Study Participants

A retrospective study of fifty-six advanced non-small cell lung cancer patients who enrolled in the Thai nintedanib Named Patient Use program from eight centers across Thailand was conducted to evaluate the treatment efficacy of combination nintedanib and docetaxel as a treatment after platinum-doublet chemotherapy during 2017-2018. These eight centers included four hospitals in Bangkok: The King Chulalongkorn Memorial Hospital, Siriraj Hospital, Ramathibodi Hospital, and Rajavithi Hospital, and four provincial hospitals: Maharaj Nakorn Chiang Mai Hospital, Chiangmai, Srinakarin Hospital, Khon Kaen Hospital, and Songklanagarind Hospital, Songkhla. This study is a collaborative project of the Thai Society of Clinical Oncology: Lung Cancer Working Group.

All patients had either a cytologic or histologic confirmed diagnosis of NSCLC. Demographic characteristics were obtained from individual patients. Treatment decision, assessments, and follow-up were obtained from individual physicians as standard practice per institute through medical records. Patient death date was validated from The Bureau of Registration Administration, Ministry of Interior, Bangkok, Thailand. This study was approved by the Ethics Committee of each local institution: Faculty of Medicine, Chulalongkorn University, Bangkok, Thailand (IRB 536/62), Faculty of Medicine, Siriraj Hospital, Mahidol University [IRB 349/2563 (EC4)], Faculty of Medicine, Khon Kaen University (HE631180), Faculty of Medicine, Ramathibodi Hospital, Mahidol University (IRB MURA2020/794), Faculty of Medicine, Chiang Mai University (IRB MED-2563-07205). Written informed consent was waived from individual study participants as permission from the director of each hospital was granted. Objective response and progression of disease were determined by local investigators using RECIST version 1.1 (13).

### Statistical Analysis

Mann-Whitney *U* test was used to assess the difference between groups of non-parametric distributed variables. Chi-square or Fisher’s exact tests were used for categorical variables. There were varying lines of combination docetaxel/nintedanib treatment from second to fourth. Then progression free survival of docetaxel/nintedanib was defined as duration from start of docetaxel/nintedanib treatment to disease progression or death. Overall disease control rate was defined as the best response evaluation of complete remission and partial response by the provided physician. We applied RECIST criteria version 1.1 as the standard oncology practice in Thailand. Overall survival was defined as the duration from diagnosis of cancer to death from any cause or at censored time which was defined on December 31, 2019. A Survival curve comparison was performed using the Kaplan-Meier method and log-rank test. The cox proportion hazards regression analysis was used to estimate multivariate hazard ratios of progression-free survival and overall survival. A two-sided *p*-value of less than 0.05 was defined as statistically significant. All statistical analyses were carried out using R version 3.3.0.

## Results

### Demographic Characteristics and Treatment Overview

From January 2017 to October 2018, 56 patients from eight centers who received combination docetaxel/nintedanib treatment in advanced stage were enrolled in this retrospective study. Patients in this retrospective cohort received nintedanib *via* Named Patient Use program following the criteria of having advanced non-small cell lung cancer with disease progression after platinum-doublet chemotherapy. Among them, 62.5% of patients were male with majority ECOG performance status of 0-1 (89.2%) and adenocarcinoma cell type (88%). Demographic characteristics and patient treatments are shown in **Tables 1**, **2**. *EGFR* and *ALK* testing were performed in 82.1% and 50%, respectively by using a standard platform of testing according to each institute. 35% had a sensitizing *EGFR* mutation that was composed of *EGFR* exon 19 deletion (n=12; 75%) and L858R (n=4; 25%). 61% of patients received more than three lines of treatment which included chemotherapy, EGFR TKI, and immunotherapy as combination or single agent. Pemetrexed, paclitaxel, and gemcitabine were commonly used as part of platinum-doublet chemotherapy at 34%, 30.3% and 23.2%, respectively. 88% of patients received 60 mg/m^2^ instead of 75 mg/m^2^ of docetaxel as the common Thai standard practice in advanced stage disease. Eight patients (14.2%) received immunotherapy as a line of standard treatment in advanced stage. Among them, four patients received immunotherapy before docetaxel/nintedanib combination treatment (**Table 2**).

**Table 1:** Demographic characteristics of participants in this study.

Characteristic		N (%)
Sex	Male	35 (62.5%)
	Female	21 (37.5%)
Age at diagnosis	< 60 years	32 (57.1%)
	> 60 years	24 (42.9%)
ECOG performance status at recurrence/metastasis	0-1	50 (89.2%)
	>2	6 (10.8%)
Smoking status	Current/Former smoker	27 (48%)
	Never smoker	29 (52%)
Histology	Adenocarcinoma	49 (88%)
	Squamous cell carcinoma	1(1.5%)
	Large cell carcinoma	1(1.5%)
	NSCLC NOS	5 (9%)
*EGFR/ALK* alteration	Exon 19 del (n=12)/L858R (n=4)	16/46 (34.7%)
	ALK overexpression	2/28 (7%)
Reimbursement	Universal/social insurance	18 (32%)
	CSMBS/state enterprise	31 (55%)
	Out of pocket	7 (13%)
Initial stage at diagnosis	Advanced stage	46 (82%)
	Relapsed/recurrence	10 (18%)
Lines of treatment	< 3 regimens	22 (39.3%)
	> 3 regimens	34 (60.7%)

^¶^ECOG performance status denotes the Eastern Cooperative Oncology Group (ECOG) scale; a performance status grade of 0 indicates asymptomatic; 1 restricted in strenuous activity but ambulatory; 2 ambulatory and capable of all self-care but unable to carry out any work activities.

**Table 2:** Summary treatment of participants in this study.

Details of treatment		N (%)
Previous platinum-doublet chemoRx regimen	Platinum-based/gemcitabine	13 (23.2%)
	Platinum-based/paclitaxel	17 (30.3%)
	Platinum-based/vinorelbine	1 (1.8%)
	Platinum-based/pemetrexed	19 (34%)
	Platinum-based/etoposide	1 (1.8%)
	Platinum-based/paclitaxel/bevacizumab	1 (1.8%)
	Platinum-based/gemcitabine/bevacizumab	2 (3.5%)
	Platinum-based/pemetrexed/bevacizumab	2 (3.5%)
Number of Rx lines before docetaxel/nintedanib	One-line	36 (64%)
	Two-lines	11 (17%)
	Three-lines	5 (9%)
Relapse-time of platinum-doublet chemotherapy	< 3 months	15 (26.7%)
	< 6 months	31 (55%)
Sequence of immunoRx	Never received immunoRx	48 (86%%)
	Before docetaxel/nintedanib	4 (7%)
	After docetaxel/nintedanib	4 (7%)

### Outcome of Platinum-Doublet Chemotherapy

The majority of patients (80%) received platinum-doublet chemotherapy as a first-line metastatic setting. Among them, five patients received bevacizumab as part of a combination and maintenance treatment. The median cycle of platinum-doublet chemotherapy was five cycles [range 1-14]. Median PFS of platinum-based doublet chemotherapy was 5.6 months [range 0.5-48, 95% CI: 4.9-6.8]. 31 patients (55%) had disease progression in less than 6 months since the start of treatment, while 15 patients (26.7%) had disease progression in less than 3 months.

### Outcome of Combination Docetaxel and Nintedanib

**Figure 1:**
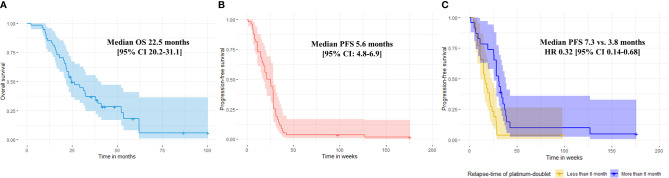
Median overall survival **(A)** and median progression-free survival of combination docetaxel/nintedanib of the entire population **(B)**. Progression-free survival of combination docetaxel and nintedanib according to relapse-time of platinum-doublet chemotherapy. Median PFS were 7.3 months vs. 3.8 months for relapse-time of platinum doublet chemotherapy ≥ 6 months vs. < 6 months, respectively (multivariate HR 0.32 [95% CI: 0.14-0.68], *p*-value 0.003) **(C)**.

The median number of treatment cycles of docetaxel and nintedanib as part of combination docetaxel/nintedanib were 6 [range 1-10] and 5 cycles [range 0-43], respectively. Overall disease control rate (DCR) of combination docetaxel/nintedanib was 57%. The median PFS was 5.6 months [range 0.25-45, 95% CI: 4.8-6.9] (**Figure 1B**) which was longer than median PFS in the LUME-lung 1 study (median PFS 3.4 months [95% CI 2.9-3.9]. Three patients (5%) stopped docetaxel/nintedanib after the first cycle due to intolerance/toxicities and response of treatment could not be evaluated. 10 patients (17%) and 20 patients (35%) had either interrupted or reduced doses of docetaxel and nintedanib, respectively. The prevalence of dose modification of nintedanib in our study was higher than the LUME lung I study (18.6%). Three patients continuing maintenance nintedanib beyond eight cycles of docetaxel and were censored on December 31, 2019.

### Analysis According to Relapse-Time of Platinum-Double Chemotherapy

The efficacy of docetaxel/nintedanib disease control was categorized by relapse-time of platinum-doublet chemotherapy i.e. rapid (less than 3 months) or slow progressor (more than 3 months). For excluded patients who could not tolerate treatment, median PFS of combination docetaxel/nintedanib for rapid relapse-time of platinum-doublet chemotherapy was 3.6 months [range 1-3; 95% CI: 2.5-5.5] which was significantly shorter than slow progressor which had a median PFS of 6.4 months [range 1.2-43.9; 95% CI: 4.9-7.4, *p*-value 0.03]. Using a relapse-time of 6 months also represented shorter disease control from combination docetaxel/nintedanib than relapse-time of more than 6 months. Median PFS for patients who had a relapse-time of platinum-doublet chemotherapy of less than 6 months and more than 6 months were 3.8 months [range 1-24.4; 95% CI: 3.2-5.2] and 7.3 months [range 1.2-43.9; 95% CI: 5.1-8.6, *p*-value = 0.01), respectively (**Figure 1C**).

### Analysis According to Sequence of Immunotherapy Treatment

The efficacy of docetaxel/nintedanib according to the sequence of immunotherapy, either before or after, was explored. Four patients who received combination docetaxel/nintedanib after immunotherapy had a median PFS of 7.9 months [range 5.2-9.1]. This duration seemed longer than the median PFS of the remaining patients (median PFS 4.5 months, range 0.25-43.9; 95% CI: 4.0-6.0, *p*-value 0.09). A response assessment was done in three patients and revealed a partial response rate of 50% and stable disease rate of 25%. One patient who received only one cycle of docetaxel/nintedanib after immunotherapy was not evaluated for response due to toxicity from treatment.

### Cox Proportional Hazards Regression Model for Prognostic and Predictive Factors

The median OS of the entire cohort was 22.5 months [range 2.2-100.1; 95% CI 20.2-31.1] (**Figure 1A**). We evaluated prognostic factors of overall survival using cox proportional hazards regression model and applied it to all potential factors including age, ECOG, smoking status, histology, oncogenic alteration, relapse-time of platinum-doublet chemotherapy, line of treatment, and sequence of docetaxel/nintedanib after immunotherapy (**Table 3**) and found that none of them prognosticated survival in our study.

**Table 3:** Univariate and multivariate analysis of prognostic factors to overall survival benefit including demographic characteristics, treatment by using Cox proportional hazards regression model.

Variables	Univariate, HR [95% CI]	*p*-value	Multivariate, HR [95% CI]	*p* value
Sex (Female vs. Male)	0.83 [0.45-1.53]	0.55	1.32 [0.34-5.13]	0.67
Age (> 60 vs. <60)	0.81 [0.45-1.48]	0.51	1.06 [0.40-2.75]	0.90
ECOG (0-1 vs. >2)	0.60 [0.25-1.43]	0.25	0.40 [0.12-1.26]	0.12
Smoking status (Never vs. Former/Current)	0.61 [0.33-1.13]	0.11	0.40 [0.12-1.37]	0.14
Histology (Adenocarcinoma vs. Non-adenocarcinoma)	0.44 [0.19-1.01]	0.05	0.47 [0.12-1.90]	0.29
*EGFR/ALK* alteration (Present vs. Absent)	0.58 [0.29-1.18]	0.13	0.79 [0.32-1.97]	0.62
Lines of treatment (> 3 vs.< 3)	0.59 [0.32-1.08]	0.08	0.64 [0.25-1.58]	0.33
Relapse-time of platinum-doublet (> 6 vs. < 6 months)	0.76 [0.42-1.38]	0.36	0.78 [0.34-1.79]	0.56
Sequence of docetaxel/nintedanib (after immunoRx vs. none)	1.26 [0.38-4.14]	0.69	3.72 [0.85-16.1]	0.07

We further analyzed predictive factors of combination docetaxel/nintedanib to define which patient subgroup might benefit most from this treatment. Nevertheless, we restricted predictive factor analysis for combination docetaxel/nintedanib in only the adenocarcinoma subtype following the USFDA approval indication. Relapse-time of platinum-doublet of more than 6 months was correlated with longer PFS with the HR of 0.36 [95% CI 0.19-0.69]. It was also an independent predictive factor of progression-free survival from docetaxel/nintedanib with the multivariate HR of 0.32 [95% CI: 0.14-0.68, *p*-value 0.003] (**Table 4**). Relapse-time of platinum-doublet chemotherapy of more than 6 months provided benefits of combination docetaxel/nintedanib treatment compared to the relapse-time of platinum-doublet chemotherapy of less than 6 months.

**Table 4:** Univariate and multivariate analyses of predictive factors of combination docetaxel/nintedanib treatment for adenocarcinoma subtype including demographic characteristics and treatment by using Cox proportional hazards regression model.

Variables	Univariate, HR [95% CI]	*p* value	Multivariate, HR [95% CI]	*p* value
Sex (Female vs. Male)	0.97 [0.53-1.80]	0.94	0.78 [0.27-2.23]	0.64
Age (> 60 vs. <60)	0.68 [0.38-1.22]	0.19	0.94 [0.43-2.07]	0.89
ECOG (0-1 vs. >2)	0.66 [0.23-1.86]	0.43	0.75 [0.24-2.39]	0.63
Smoking status (Never vs. Former/Current)	0.74 [0.41-1.34]	0.33	0.73 [0.27-1.96]	0.54
*EGFR/ALK* alteration (Present vs. Absent)	1.31 [0.67-2.57]	0.42	1.14 [0.53-2.44]	0.72
Relapse-time of platinum-doublet (> 6 vs. < 6 months)	0.36 [0.19-0.69]	0.001*	0.32 [0.14-0.68]	0.003*
Sequence of docetaxel/nintedanib (after immunoRx vs. none)	0.50 [0.15-1.65]	0.26	1.05 [0.30-3.72]	0.92

*Statistically significant.

## Discussion

Novel strategies to define treatment by adopting predictive biomarkers are currently accepted as standard practice. However, in the setting of subsequent treatment after disease progression, there are limitations of biomarker usage. Subsequent immunotherapy after platinum-doublet chemotherapy were explored in several randomized phase III trials to improve patient survival benefit and ensure quality of life (5, 14, 15). There was more progression disease and shorter PFS compared to the standard treatment arm of docetaxel. This could imply that a novel immune checkpoint inhibitor did have efficacy of long term durability and disease control in a limited number of patients in a second-line setting (11). Adding anti-angiogenesis such as nintedanib to docetaxel is another option that has been approved by the USFDA as second-line treatment after platinum-resistance in advanced NSCLC patients with adenocarcinoma subtype (2). However, there is no comparative efficacy of this combination to immunotherapy. The strategy to enhance treatment efficacy by modulating the sequence of treatment requires further elucidation. There are potential high objective response rates and PFS reports for the nintedanib/docetaxel treatment combination after immunotherapy in a case series from the Spanish Named patient used program (ORR 36%, median PFS 3.2 months [95%CI: 1.4-14.6]) (16) and the prospective non-interventional VARGADI cohort study (ORR 58%, PFS 5.5 months [95% CI: 1.9-8.7]) (10). In our series, albeit a small sample size to validate the results, the median PFS for patients who received combination nintedanib/docetaxel after immunotherapy (median PFS 7.9 months, range 5.2-9.1) was longer than the rest of the patients in this retrospective cohort (median PFS 4.5 months, range 0.25-43.9; 95% CI: 4.0-6.0, *p*-value 0.09). Among the adenocarcinoma subtype, the Cox proportional hazard regression analysis did not indicate superiority of sequential docetaxel/nintedanib after immunotherapy in terms of PFS with HR 0.50 [95% CI: 0.15-1.65, *p*-value 0.26] by univariate analysis and HR 1.05 [95% CI: 0.30-3.72, *p*-value 0.92] by multivariate analysis.

Advanced stage adenocarcinoma histology lung cancer patients who had rapid progression of platinum-doublet chemotherapy within 9 months had better outcomes when adding nintedanib to docetaxel as a subsequent treatment, which can be translated to survival outcome (17, 18). Advanced stage non-small cell lung cancer with rapid progressive disease might not be fruitful for immunotherapy (19). However, none of our patients who received immunotherapy had rapid progression from platinum-doublet chemotherapy. This limits our ability to explore this issue. Furthermore, there was a smaller proportion than the general prevalence of sensitizing *EGFR* mutation in this cohort (34.7%) which represented physician selections of preferred non-oncogenic addicted advanced stage lung cancer for anti-angiogenic treatment. However, among the adenocarcinoma subtype, neither *EGFR* nor *ALK* alteration impacts the outcome of this combination. A relapse time of platinum-doublet chemotherapy of more than 6 months solely indicated the benefit of combination docetaxel/nintedanib treatment compared to the relapse time of platinum-doublet chemotherapy of less than 6 months by multivariate HR of PFS 0.32 [95% CI: 0.14-0.68, *p*-value 0.003].

We would like to declare our study limitations. First, the retrospective cohort prohibits us to retrieve complete information, for example, toxicity of treatment and precise time of imaging evaluation. There might be variations among each center that provided treatment for patients. The treatment lines of combination nintedanib/docetaxel varied from second to forth line. Heavy pretreatment chemotherapy might evolve resistance clones than limited-line treatment. Moreover, the median PFS of combination docetaxel/nintedanib in our study (5.6 months, 95% CI 4.8-6.9) was longer than the LUME lung-1 study (3.4 months, 95% CI 2.9-3.9). The higher frequency of imaging evaluation in LUME lung-1 [first at 4-weeks and then every 6- weeks after randomization compared to our usual standard practice (every 9-weeks)] probably explains this finding. Lastly, even though the general global recommended dosage of docetaxel is 75 mg/m2, in Thailand, the standard used is 60 mg/m2. No direct comparison between dosage efficacy has been reported, however, results from a prospective randomized phase IIb (SENECA study) revealed less toxicity, such as afebrile neutropenia and mucositis of combination nintedanib/docetaxel from the lower dosage (33 mg/m^2^ D1,D8) of docetaxel, without any compromise of efficacy (20).

## Data Availability Statement

Due to confidentiality agreements, supporting data can only be made available to bona fide researchers subject to a non-disclosure agreement. Please contact the corresponding author (chanida.vi@chula.ac.th).

## Ethics Statement

This study was approved by the Ethics Committee of each local institution: Faculty of Medicine, Chulalongkorn University, Bangkok, Thailand (IRB 536/62), Faculty of Medicine, Siriraj Hospital, Mahidol University (IRB 349/2563 (EC4)), Faculty of Medicine, Khon Kaen University (HE631180), Faculty of Medicine, Ramathibodi Hospital, Mahidol University (IRB MURA2020/794), and Faculty of Medicine, Chiang Mai University (IRB MED-2563-07205). Written informed consent for participation was not required for this study in accordance with the national legislation and the institutional requirements.

## Author Contributions

Conception and design: CV. Administrative support: CV. Provision of study materials or patients: KK, PD, TR, BC, JC, KM, CS, and LT. Collection and assembly of data: KK and CV. Data analysis and interpretation: CV. Manuscript writing: all authors. Final approval of manuscript: all authors. All authors contributed to the article and approved the submitted version.

## Funding

This work was supported by Thai Society of Clinical Oncology: Lung Cancer Working Group.

## Conflict of Interest

The authors declare that the research was conducted in the absence of any commercial or financial relationships that could be construed as a potential conflict of interest.

## Abbreviations

FDA, Food and Drug Administration; MDSC, Myeloid-derived suppressor cell; VEGFR, Vascular endothelial growth factor receptor.
