# Supplementary material for: Efficacy of Combination Docetaxel and Nintedanib in Advanced Non-Small Cell Lung Cancer in Thailand: A Multicenter Study
Source: Front Oncol. 2021 Apr 29;11:572740. doi: 10.3389/fonc.2021.572740 (PMC8117590; doi:10.3389/fonc.2021.572740)
Supplement: Supplementary file 1 [file DataSheet_1.docx]

**Efficacy of combination docetaxel and nintedanib in advanced non-small cell lung cancer in Thailand; a multicenter study**

Krittiya Korphaisarn MD.^1**^, Pongwut Danchaivijitr MD.^1**^, Thanyanan Reungwetwattana MD.^2^, Busayamas Chewaskulyong MD.^3^, Luangyot Thongthieang MD.^4^, Jarin Chindaprasirt MD.^5^, Kunlatida Maneenil MD.^6^, Chirawadee Sathitruangsak MD.^7^, Chanida Vinayanuwattikun, MD., Ph.D.^8*^

Thai Society of Clinical Oncology : Lung Cancer Working Group

**represented co-first author

*represented corresponding author

**Author’s affiliations**

^1^Division of Medical Oncology, Department of Medicine, Faculty of Medicine Siriraj Hospital, Mahidol University, Siri Rat, Bangkok Noi, Bangkok 10700, Thailand

^2^Division of Medical Oncology, Department of Medicine, Faculty of Medicine Ramathibodi Hospital, Mahidol University, 270 Rama 6 Road, Tungpayathai, Ratchathewee, Bangkok 10400, Thailand

^3^Division of Medical Oncology, Department of Medicine, Faculty of Medicine, Chiangmai University, Tambon Su Thep, Mueang Chiang Mai District, Chiang Mai 50200, Thailand

^4^Division of Oncology, Department of Medicine, Faculty of Medicine, Khon Kaen Hospital. Khon Kaen University, Khon Kaen 40000, Thailand

^5^Department of Medicine, Faculty of Medicine, Srinagarind Hospital, Khon Kaen University, Khon Kaen, 40002, Thailand

^6^Medical Oncology Unit, Department of Medicine, Rajavithi Hospital, Bangkok, Ratchathewi District, Bangkok 10400, Thailand

^7^Holistic Center for Cancer Study and Care (HOCC-PSU) and Division of Medical Oncology, Department of Internal Medicine, Faculty of Medicine, Prince of Songkla University, Hat Yai, Songkhla, 90110, Thailand

^8^Division of Medical Oncology, Department of Internal Medicine, Faculty of Medicine, Chulalongkorn University and the King Chulalongkorn Memorial Hospital, Bangkok, 10330, Thailand

**Supplementary information**

**Outcome of immunotherapy**

Eight patients (14.2%) received immunotherapy composed of nivolumab (50%) and pembrolizumab monotherapy (25%) as a line of treatment. Two patients who received pembrolizumab as combination therapy with pemetrexed had stable disease as maximal response assessment. Disease control periods for combination treatment were 6.3 and 10.5 months. The remaining 6 patients who received single agent immunotherapy had a median PFS of 2.9 months [range 1.5-16.2]. Maximal response assessment was missing in 1 patient who received only one cycle of treatment while in the remaining 5 patients, there were 3 with stable disease (60%), 2 with progression (40%).

**Outcome of tyrosine kinase inhibitor**

Sixteen patients (28.5%) received EGFR tyrosine kinase inhibitor (afatinib 3, erlotinib 6 and gefitinib 7). Median PFS was 12.5 months [range 0.3-49.3; 95% CI: 7-24.8]. One patient who had shortest disease control 1.4 weeks was not tested for *EGFR* mutation. One patient with *EGFR* 19 deletion had *de novo* EGFR TKI resistance. This patient also had rapid relapsed-time (<3 month) of platinum-doublet chemotherapy. Two patients who had positive ALK expression received first-generation and subsequent second generation ALK tyrosine kinase inhibitor; crizotinib and ceritinib, respectively. Both continued on ceritinib without disease progression and were censored at December 31, 2019. Disease control in those 2 patients by combining 2 regimens of ALK tyrosine kinase inhibitor were 28.7 and 16.2 months.
